# Observations on the Causes and Prevention of Premature Delivery and Death of the Fœtus

**Published:** 1830-04

**Authors:** James Russell

**Affiliations:** Surgeon and Accoucheur.


### PREMATURE DELIVERY.

*Observations on the Causes and Prevention of Premature Delivery and Death of the Fœtus.* By James Russell, Surgeon and Accoucheur.

Every practitioner in midwifery must have met with numerous patients who have been delivered in consequence of the death of the fœtus having occurred during the progress of utero-gestation. He must also have been struck with the remarkable circumstance, that the same patient has been repeatedly delivered of premature children, mostly stillborn, and at nearly the same period, where the state of health of the patient could not account for the event. These circumstances occur with a degree of frequency which can scarcely be suspected by those not conversant with the practice of midwifery: they are of constant occurrence in extensive practice in that department of the profession.

The cases to which these observations are intended to apply are, where the death of the fœtus takes place after the twentieth week of utero-gestation, without any direct or evident cause; and to the premature birth of unhealthy children, more particularly where either of these occurrences is repeated in subsequent pregnancies.

It will afford no small gratification to the medical inquirer to learn that there exists a certain and simple remedy for these affections, which has succeeded with a uniformity of result not usual in the ordinary effects of medical agents. The remedy which has been found so successful is mercury,* continued for a considerable period; and the conclusion which led to this practice was the conviction that all these cases depended on the syphilitic poison, and were curable by the same means.

In the early part of the writer's professional career, his attention was called to some strongly marked cases, where the influence of syphilis was clearly traced, and its effects upon the fœtus unequivocally ascertained. The striking analogy between the clearly marked and other cases which occurred about the same time, but where this disease could not be so traced, led him to adopt this mode of practice; the success of which has been confirmed by continued experience in numerous cases, with one uniformly successful result.

There is no fact better known in medicine than that of the influence of the venereal disease in destroying the life of the fœtus in utero.† It would be needless to call the

* It is remarkable that, Dr. Colson formerly house-surgeon of the Hôpital des Veneriens, published, some time ago, in the Archives Générales, an elaborate paper, for the purpose of showing that menorrhagia, amenorrhœa, and *abortion*, are very frequently *caused by the influence of mercury*. Dr. Colson illustrates his opinion by a series of cases, which certainly appear to bear him out in his inference. He enumerates six cases where the *premature expulsion of the fœtus* was apparently referrible to the action of mercury. Dr. C. remarks, that miscarriages are very common in the venereal hospitals at Paris, though this accident is usually attributed to the syphilitic complaint, and not to the mercury employed for its cure. He is of a different opinion, and attributes the abortions to the mercury: he supports this opinion by various arguments. We have frequently exhibited mercury to pregnant women, for syphilis and other diseases, and no facts have occurred to our observation tending to provê that this remedy, when *cautiously* employed during pregnancy, at all interfered with the course and natural duration of utero-gestation, or the health of the infant.—Editor.

† The following extract from Mr. Bacot's Treatise on Syphilis, p. 254, is directly in point, and confirms the efficacy of Mr. Russell's practice:

"Mr. Hey mentions a circumstance (Med. Chir. Trans. vol. 7,) which has lately been insisted upon more forcibly by Dr. O'Brien; it is this: a woman shall repeatedly miscarry at the end of the seventh or eighth month, and be delivered of a dead child: or, if the child be born alive, it exhibits all the appearances of disease, and soon dies. Dr. O'Brien, whose paper is published attention of the medical profession to the fact of mercury remedying this state, it being already perfectly understood. The extent, however, of the influence of this poison on the life and health of the fœtus has not yet been fully investigated, and a further inquiry into its effects becomes of great practical importance. It is desirable to ascertain whether its influence is not greater than is generally suspected. The object of these observations is to call attention to this subject, and to the important fact that almost every case of the description before mentioned is remedied by the same means as the cases clearly traced to the syphilitic poison.

The circumstance of mercury being so beneficial in every case of this nature, and the similarity of symptoms, lead to a well-grounded suspicion that they have a common origin, and that, however difficult it may be to trace the poison, it must nevertheless have existed at some former period in one of the parents. The uniform success of mercury can only be accounted for on the supposition that this affection depends on the same disease which is cured by this remedy. It is one of the few specifics we possess; and we must either infer that it has additional specific properties besides those it exerts in syphilis, or that its uniform efficacy depends on its remedying an effect of this disease. It is more difficult to procure correct information on this point than any other in the whole range of medical inquiry. The parents have probably been many years beyond suspicion and in perfect health. An inquiry of this nature requires to be conducted with a great degree of caution, for even a slight insinuation might entirely destroy the peace of a family, as nothing is more likely to distress the mind of either parent than the in the Trans. Coll. Phys. Dublin, relates many cases of this kind, and assures us that he has in several instances of this kind been led to the belief of a syphilitic taint lurking in the constitutions of the mothers, and that his suspicions have been confirmed by the result of a course of mercury, which has not only restored the health of the patients (previously valetudinary), but has enabled them to become mothers of living and healthy children. I cannot say that I have any evidence of my own to offer on this subject. I mention the facts as related by the above respectable authorities, and it will be worth the young surgeon's attention, in after-life, to bear in mind what they have urged, should such or similar instances come under his observation. Certainly where a female, formerly in good health, has declined without apparent reason, where she is repeatedly miscarrying at one particular period of her pregnancy, or bringing forth puny children, dying almost as soon as they come into the world, it may not be amiss to turn our attention to the probably syphilitic source of her misfortune, and, by gentle beginnings, to try the effect of a mercurial treatment; and, if the above authors are to be believed, the result will be most satisfactory."—Editor.

supposition that a cause of this kind has blighted their hopes of offspring, and rendered them childless.

Little dependence can therefore be placed on direct evidence respecting the previous state of health of the parent most liable to suspicion: it is evident, however, that there exists, in most instances, no other suspicion of syphilitic poison than this state of the fœtus, and we are induced to argue its existence from the strong analogy in the symptoms and the effects of mercury already detailed. If this correspondence in symptoms and remedy should be admitted as proofs of its existence, it proves the remarkable fact of its existing for many years lurking in the constitution, and may lead to important conclusions in the treatment of this disease, as far as depends on a more continued use of mercury.

The writer does not seek that any higher authority should be given to his opinions than what is drawn from the analogy of symptoms and the success of the treatment; for he must confess that he has been deterred from minute investigation by the reasons before mentioned, and has pressed his inquiries less strictly than he should have done had he considered this distinction necessary to the success of his practice. The assertion, therefore, of the common origin of all cases of this nature requires further practical investigation.

Whatever opinion may be formed as to the correctness of the theory, it is of less importance than the fact that all these cases are remedied by one common remedy; and this statement rests on very different grounds from the opinion respecting their origin. It has been founded not on reasoning, but on continued experience in numerous cases, although it will not be disputed but that cases may occur where the remedy will fail; yet the writer has hitherto met with no instances of this kind, and he unequivocally declares that every case of this nature which he has hitherto met with, where the remedy has been fairly administered, has been followed by success.

It may be inferred that this favorable result might have followed had no mercury been administered, and that the patient, having had one child born under such circumstances, would have had a healthy child at the full period without its aid; but it has so happened that almost every case in which it has been administered has been where the patient has had more than one premature labour, with similar symptoms in each; clearly showing that there was some cause in operation independent of accident to produce this effect. Many of the patients had not been seen until after repeated premature births, others had neglected the precautions until the same result proved to them the necessity of attending to the directions they had received, and others again neglected these directions from probably the unworthy motive of being freed from the trouble of a family.

There are two kinds of this morbid affection: one where the fœtus is stillborn, and has been dead some time; the other where the fœtus has been born alive.

The first affection never occurs before the twentieth week, but may supervene at any subsequent period of utero-gestation. There are no precise symptoms which indicate the probable occurrence of this diseased state of the fœtus. The patient, however, usually suffers more from those anomalous symptoms which accompany pregnancy; there frequently appears more general derangement of the system, and the complexion is often paler and more sallow than in ordinary cases. The most frequent period for the appearance of this state is a little before or about the seventh month. The patient, having suffered more or less as above stated, feels the flaccidity of the breast, the sinking and coldness of the abdomen, and the loss of motion, which indicate the death of the fœtus: these symptoms may remain stationary for one or two, or even three, weeks before labour comes on, but it mostly succeeds in about a week or ten days after these indications have appeared. The liquor amnii is generally in greater quantity than ordinary, which often makes the patient appear far advanced in pregnancy, or gives rise to the suspicion of twins. The sanguineous discharge during labour, and the lochia, are generally less in quantity than in natural birth. At whatever period of utero-gestation this affection occurs, the symptoms are nearly the same; and if this state be not arrested, the patient who has been delivered at one time will generally be delivered in a subsequent pregnancy, with the same series of symptoms occurring at the same period. The fœtus having in most cases been dead many days, prevents any minute appearances being exhibited beyond the ordinary signs of putrefaction, which necessarily ensue from the long retention after death. There is in these instances no appearance of the child which can enable us to distinguish this state from the appearances exhibited in death arising from evident causes, and where the fœtus has been retained equally long in the uterus.

There is another state of the fœtus in utero which is more rare, but, however, is not peculiar to this affection; for these appearances were seen in a singular case, where a patient, between seven and eight months advanced in pregnancy, had the regular pains of labour for some hours, and they then subsided. She was subsequently delivered at the full period of a healthy child, and of another which exhibited the appearances about to be described. The fœtus, instead of the usual large proportion of liquor amnii, is surrounded by a small quantity of a yellow, semi-opake, and inodorous fluid, having much the appearance of turbid bile; the limbs and body of the fœtus are remarkably shrivelled, of a whitish appearance, and without any smell. This state appears to be the commencement of the conversion of the fœtus into adipocire.

There are other cases, again, where the child appears to have been alive up to the time or nearly the time of birth, and where the putrefactive process has not begun, the skin of the extremities will, however, be found to peel off more readily than might be expected in children, whose death has taken place just before or during birth.

The second description of cases is where the child is born prematurely, and lives either a short time or survives to a considerable period: these premature births may also take place at any time after the twentieth week; but when this occurs before the seventh month, as might be expected, the child merely gasps for a short time, and then expires. Where utero-gestation has continued to the period of seven months, the child may survive for a considerable time, but most frequently dies in a few hours. In many of these instances it will be found, on examination of the body, that the skin of the hands and feet is raised in distinct round vesications, from which the skin peels off on the slightest touch; the body, and more particularly the abdomen, will be found, on close examination, to be covered with spots, but without vesication. These are likely to escape notice, unless the body is washed with a warm sponge, or has been placed in a warm bath. Where this affection of the skin exists, it can scarcely be doubted that this state of the fœtus has proceeded from a venereal taint, the eruption having much the appearance of venereal eruptions.

In other cases, again, the child is born about the seventh month, or a little later, but presents no unusual appearance, except being attenuated and weakly. It generally puts on early the appearance of wrinkled old age, and sinks from marasmus, or from the first infantile disorder by which it is attacked. It is with some difficulty that this kind is recognised as belonging to this class of affections, and it is often only discovered by repeated pregnancies occurring with similar appearances. The effects of mercury are as decidedly beneficial in this as in the other cases described.

Cases of death of the fœtus, or premature labour, occurring from other sources, may, in almost every instance, be traced to some evident cause; to accident, fright, illness of the mother, or malposition of the child or placenta: they are usually preceded by severe pains commencing some time before actual labour, by (in many cases) considerable or even severe hemorrhage, and the fœtus is rarely dead for any long period.

It is likewise important to distinguish between these cases and ordinary abortion, occurring between the eighth and sixteenth week. Although patients may miscarry repeatedly at the same period of gestation, it by no means follows that the abortion proceeds from this source, or possesses the characters here described. No instances of this affection have occurred until after the time a patient is considered safe from abortion, and the symptoms are in other respects totally dissimilar.

It would be tedious to enumerate the various cases in detail: the most frequent kind is where a patient has been delivered twice, at the same period of gestation, of a stillborn fœtus, and has subsequently been delivered of a fine healthy child at the full period. In some instances, patients have been delivered four times of children, each time in the same state, and at the same period of gestation. From the use of mercury, they have the fifth time been delivered of fine healthy children at the full period. Two cases of this kind have very recently occurred: one had always been delivered at seven months, the other at nine; with both, the children had always been stillborn.

Mercury has been administered in the same manner in almost every case: the use of it has been commenced when the patient has been about three months advanced in pregnancy; from five to eight grains of the Pil. Hydrarg. have been given twice ~~or three times~~ a day, and has been continued for about six weeks, diminishing the quantity as soon as the patient has complained of a little unpleasant taste in the mouth. It is desirable to produce this effect, but it is necessary to avoid carrying it further. At the termination of this period, the medicine has been discontinued for a month, then resumed for four or five weeks, again discontinued for a month, and then resumed for four or six weeks after the seventh month.

It is not intended to affirm that this quantity is absolutely essential, nor has it been attempted to ascertain the smallest quantity necessary. The operation of the medicine is attended with so little inconvenience, and so commonly answers the purpose of the purgatives usually required, that the patient imagines the medicine has no other effect; her health and appetite often improve under its use. It is, however, necessary to prevent the irritation of the bowels which mercury so often induces, and to combine it with more or less opium where this effect is produced. One instance occurred where the patient having taken mercury after two premature births, and of dead children, was delivered of a full-grown live child. She omitted the medicine in her fourth pregnancy, and the unfavorable result again followed; in her fifth pregnancy, she resumed the use of mercury, with the usual effect. It will, therefore, be proper to continue the use of this remedy in each subsequent pregnancy, although perhaps not to the same extent.

It will be perceived that the foregoing observations apply to a large majority of the cases of death of the fœtus in utero, and a great proportion of the cases of premature labour which occur in practice. The subject thus becomes of considerable interest. If an estimate may be formed from the number of cases of this description which are seen by individual practitioners, the whole number which occur in this metropolis alone must be very great. Should the efficacy of this remedy, and the extent of its application, be confirmed by the general experience of the profession, it will lead to a most important preservation of human life.

It too often happens that a remedy which appears to have succeeded with the individual who recommended it, with surprising efficacy, loses all its reputation in the hands of others. On this ground it must necessarily be expected that the confident assurance respecting the nature of this affection, and the remedy for its removal, will be received with considerable suspicion; but the subject is one of easy investigation.

The uniformity of treatment, and the assurance of success, will, if the observations are correct, at once enable the subject to receive that confirmation by the experience of others which is necessary to produce public confidence, and render it an established mode of treatment.

*Broad street, Golden square.*

