# Erratum

**Published:** 1830-05

**Authors:** 


ERRATUM in the last Number, p. 305. Mr. Russell requests us to correct the following passage: For "Five or eight grains of the Pil. Hydrarg. have been given twice or three times a day," read "twice a day."

